# Comparative Study of the Management of Intertrochanteric Fracture Femur With Proximal Femoral Nail vs. the Dynamic Hipscrew With Derotation Screw in Elderly Population

**DOI:** 10.7759/cureus.19431

**Published:** 2021-11-10

**Authors:** Daljinder Singh, Akashdeep Singh, Gitesh Singh, Manjit Singh, Annie Sandhu, Kuldip S Sandhu

**Affiliations:** 1 Department of Orthopedics, Government Medical College, Patiala, IND; 2 Department of Orthopedics, Gian Sagar Medical College, Mohali, IND; 3 Emergency Department, All India Institute of Medical Sciences, Rishikesh, IND

**Keywords:** lower limb trauma, dynamic hip screw fixation, proximal femoral nail, complex fracture, femur intertrochanteric fracture

## Abstract

Background: Intertrochanteric fractures can be treated, both by conservative and operative methods depending upon the status of the patient. The purpose of this study was to assess the functional outcome of intertrochanteric fracture of femur treated with dynamic hip screw (DHS) with de-rotation screw comparing and proximal femoral nail (PFN).

Methodology: We compared 30 (male: 23, female: seven) cases of intertrochanteric fractures with a mean age of the population was 65 years and male to female ratio in was 2.75:1. Patients were recruited in this study having inclusion criteria of adults above 50 years of age, isolated intertrochanteric fractures of the AO Foundation/Orthopaedic Trauma Association (AO/OTA) type A1 and A2, fracture less than two weeks, and intertrochanteric fracture with or without distal extension.

Results: Post-operatively, patients treated by either of these two methods were statistically analyzed in terms of comparing advantages and disadvantages in terms of the time of fracture union and outcome of both above-mentioned procedures using Harris hip score.

Conclusion: PFN gives better results than DHS with De-Rotation Screw-in intertrochanteric fractures in terms of the amount of blood loss during surgery, duration of surgery, early toe-touch weight-bearing, and Harris hip scores. There is no difference between the two modalities in terms of duration of hospitalization, fracture union, mortality and morbidity, and postoperative complications.

## Introduction

Fractures of the proximal femur and hip are relatively common injuries in adults. Several epidemiological studies have suggested that the incidence of fractures of the proximal femur is increasing. Intertrochanteric fracture is one of the most common fractures of the hip, especially in the elderly. The incidence of intertrochanteric fracture is rising because of the increase in the number of elderly populations superadded with osteoporosis. These fractures are three to four times more common in women, and the mechanism of injury is usually due to low-energy trauma like a simple fall [1]. Males lesser than 40 years of age contain a higher possibility of high-energy trauma hip fractures, although these are relatively rare.

Gullberg et al. [2] estimated that the future incidence of hip fracture worldwide would double to 2.6 million by 2025 and 4.5 million by 2050. The percentage increase will be greater in men (310%) than women (240%). Hagino et al. [3] reported a lifetime risk of hip fractures for individuals at 50 years of age of 5.6% for men and 20% for women.

In extra-capsular (per-trochanteric and intertrochanteric) fractures, since the fracture surface is cancellous, the union is the rule, and non-union is very rare [4]. The unique anatomical feature of the proximal femur creates a very different biomechanical environment. Re-establishing medial cortical stability is essential for lateral plate fixation, and it prevents complications of implant failure with internal fixation [5]. The unique biomechanical environment also favors intramedullary fixation compared to the extra-medullary fixation, as the former device helps to decrease the moment.

Intertrochanteric fractures can be treated, both by conservative and operative methods depending upon the status of the patient [6]. Conservative management is directly proportional to the mortality rate [6]. In 1902, Whitman re-evaluated the role of conservative treatment and advocated reduction and stabilization with traction, abduction, and internal rotation [7].

Operative treatment with internal fixation of the fracture lessens the frequency of life-threatening complications [8]. The choice of implant is mainly determined by the fracture pattern (stable or unstable). Out of various fixation devices, the dynamic hip screw is a much appropriate technique for the management of intertrochanteric fracture femur [9].

In intertrochanteric fractures, the use of DHS is seen to be associated with excessive collapse and unwanted rotation of proximal fragments leading to high failure rates [10]. A de-rotation screw can be used to counteract rotation and instability in the fractured intertrochanteric proximal fragment. If rotational stability is required in DHS, a cannulated screw is inserted above and parallel in both planes to DHS as a de-rotation screw [10]. The biomechanical advantage of intramedullary devices is important, particularly in unstable trochanteric fractures [11]. 

Aims and objectives

We studied 30 cases of intertrochanteric fracture femur patients more than 50 years old, which were managed with a proximal femoral nail or dynamic hip screw with a de-rotation screw. The patients were divided into two groups randomly, i.e., Group A and Group B, with 15 cases in each using the chit and box method. Group A contains patients treated with PFN, and Group B contains patients treated with DHS with de-rotation screw, and results were evaluated with the following objectives:

1. To compare the advantages and disadvantages of with proximal femoral nail and dynamic hip screw with de-rotation screw-in AO/OTA type A1 and A2 intertrochanteric fracture femur [12,13].

2. To compare the time of fracture union and outcome of both above-mentioned procedures using Harris hip score. 

## Materials and methods

Taking into account an alpha error of <0.05 and power of 0.8, a sample size calculation determined that a total of 27 patients was required in each group. In order to compensate for dropouts, patients were allocated into two groups of 30 participants each using a randomized selection method. Therefore, the present study was conducted on 30 cases of intertrochanteric fractures femur above the age of 50 years from November 2019 to October 2020. Ethical committee approval was taken vide no. BFUHS/2K19p-TH/11995 dated 09/10/2019.

Inclusion criteria: 1) Adults above 50 years of age. 2) Isolated intertrochanteric fractures of AO/OTA type A1 and A2. 3) Time of fracture less than two weeks. 4) Intertrochanteric fracture with or without distal extension.

Exclusion criteria: 1) Any open injury. 2) Fracture associated with neurovascular injury. 3) Polytrauma patient. 4) Patients who refuse to give consent. 5) Pathological fractures. 6) Time of fracture more than two weeks. 7) Patients having re-injury at old intertrochanteric fracture site. 7) Non-union or implant failures. 8) Pre-existing neuromuscular disease. 9) Local sepsis.

Operative management: proximal femoral nailing: the patient was put supine on the traction table, and the fracture was reduced under the image intensifier or fluoroscope. In AP view, nail entry was done on the tip or slightly medial to the tip of the greater trochanter in the curved extension of the medullary cavity, and a guidewire was inserted with the help of cannulated awl, and the cannulated drill bit was used through protection sleeve over the guidewire, and sequential reaming was done manually. The nail was carefully inserted into the femoral opening with slight twisting movements, and proximal and distal locking was done.

Dynamic hip screw with de-rotation screw: the fracture site was exposed using lateral incision by splitting vastus lateralis sub-periosteally. Guidewire was passed in the dead center of the head and neck in both AP and lateral views using the 135-degree angle guide till the “bulls-eye” or center-center placement was achieved.

De-rotation lag screw: additional lag screw was inserted at this point to prevent the rotation of the proximal fragment, and a sliding hip screw was inserted within 10 mm of subchondral bone.

Postoperative and follow-up: postoperatively patients were followed up for radiological evaluation to ascertain union, implant position, any sign of implant failure, and loss of reduction at monthly intervals for six months, then after three-month intervals till union. As per Harris Hip Score, the result is considered successful if there is a postoperative increase in Harris Hip Score more than 20 points and the implant is radiologically stable.

## Results

The mean age of the population was 65 years (Range from 52 years to 71 years), and the male to female ratio in this study was 2.75:1. In our study, more patients were recruited between 52-60 years as a result of 1) preference of more elderly to opt for conservative management and 2) decreased surgical procedures over elderly due to co-morbidities. In the current study, the difference in the ratio of incidence is due to 1) more active lifestyle of males and 2) females are mostly confined to households.

Among both groups, fall and Road Traffic Accident (RTA) were the modes of trauma among nine and six patients in group one, respectively. In the PFN group, fall and RTA were responsible for trauma in 12 and three patients, respectively. 

Among the patients of the DHS with de-rotation screw group, nine patients and six patients belonged to the category A1 and A2, respectively. Among the patients of the PFN group, seven patients and eight patients belonged to the category A1 and A2, respectively.

In the DHS with de-rotation screw group, the union was observed on radiological examination after 12 weeks in four (26.7%) patients, while the minimal union was observed in 11 (73.3%) patients. The PFN union was observed in nine (60%) patients, and the minimal union was observed in six (40%) patients. In group one, callus was observed on radiological examination after 24 weeks in 11 patients, and adequate callus (union) was observed in four patients. In group two, callus was observed in six and adequate callus (union) was observed in nine patients (Table 1).

**Table 1:** Radiological union at 24 weeks DHS with DRS: dynamic hip screw with de-rotation screw, PFN: proximal femoral nail

Radiological Union at 24 weeks	DHS with DRS Group		PFN Group	
	Patients	Percentage	Patients	Percentage
Adequate callus seen	4	26.67%	9	60%
Callus seen	11	73.33%	6	40%
Total	15	100%	15	100%
X^2^	3.28			
p-value	0.065 (NS)			

Lag screw cut-out and “Z” effect were observed in one patient in each group. In group one, diabetes and hypertension were observed in one and three patients, respectively, and in the PFN group, diabetes was observed in two and hypertension in three patients (Table 2).

**Table 2:** Complications DHS with DRS: dynamic hip screw with de-rotation screw, PFN: proximal femoral nail

Complications	DHS with DRS Group		PFN Group	
	Patients	Percentage	Patients	Percentage
De-rotation and lag screw cut out	1	6.67%	0	0%
Z effect	0	0%	1	6.67%
None	14	93.33%	14	93.33%
Total	15	100%	15	100%
X^2^	0.54			
p-value	0.464			

**Figure 1:**
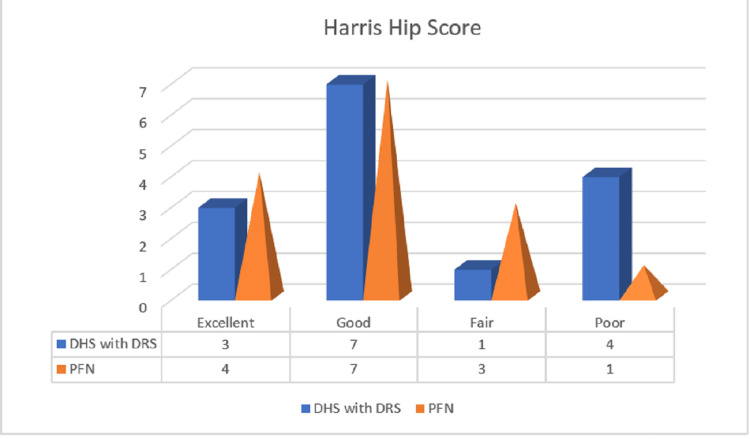
Comparison of interpretation of mean Harris Hip Score among groups one and two DHS with DRS: dynamic hip screw with de-rotation screw, PFN: proximal femoral nail

Mean Harris hip scores among the patients in groups one and two were found to be 73.6 and 88.1, respectively (P-Value > 0.05) (Table 3, Figure 1).

**Table 3:** Comparison of mean Harris hip score DHS with DRS: dynamic hip screw with de-rotation screw, PFN: proximal femoral nail

	Group	N	Mean	SD	Std. Error Mean	t-test	p- value
HARRIS HIP SORE	DHS with DRS	15	78.93	8.51	2.20	1.390	0.175
	PFN	15	82.67	5.98	1.55		

 

Mean blood loss among groups one and two were found to be 312 and 108 ml, respectively (P-Value < 0.05). The mean duration of surgery in the patients of group one and group two were found to be 48.8 and 38.6 minutes, respectively (P-Value < 0.05) (Table 4). Meantime of partial weight-bearing in groups one and two were found to be 6.13 and 6.2, respectively (P-Value > 0.05) (Table 4). Meantime of full weight-bearing in groups one and two were found to be 14.13 and 14.2, respectively (P-Value > 0.05) (Table 4).

**Table 4:** Mean value, standard deviation, and P-value of various descriptive parameters DHS with DRS: dynamic hip screw with de-rotation screw, PFN: proximal femoral nail * (Significant)

	_Groups _	_Number_	_Mean _	_SD _	_Std. _ _Error _ _Mean _	_t-test _	_p-value _
_Duration of s__urgery _ _(minutes) _	_DHS with _ _DRS _	_15 _	_48.80 _	_4.07 _	_1.05 _	_5.789 _	_0.001*_
	_PFN _	_15 _	_38.60 _	_5.47 _	_1.41 _		
_Total a__mount of b__lood loss_ _(ml) _	_DHS with _ _DRS _	_15 _	_312.00 _	_30.28 _	_7.82 _	_24.958 _	_0.001*_
	_PFN _	_15 _	_108.00 _	_9.22 _	_2.38 _		
_Duration of h__ospital s__tay after s__urgery _	_DHS with _ _DRS _	_15 _	_10.80 _	_1.47 _	_0.38 _	_1.841 _	_0.076_
	_PFN _	_15 _	_9.47 _	_2.39 _	_0.62 _		
_Toe-touch w__eight b__earing _ _(days) _	_DHS with _ _DRS _	_15 _	_6.87 _	_1.19 _	_0.31 _	_1.993 _	_0.056 _
	_PFN _	_15 _	_5.80 _	_1.70 _	_0.44 _		
_Partial w__eight b__earing _ _(weeks) _	_DHS with _ _DRS _	_15 _	_6.13 _	_1.36 _	_0.35 _	_0.127 _	_0.900 _
	_PFN _	_15 _	_6.20 _	_1.52 _	_0.39 _		
_Full weight b__earing _ _(weeks) _	_DHS with _ _DRS _	_15 _	_14.13 _	_1.36 _	_0.35 _	_0.127 _	_0.900_
	_PFN _	_15 _	_14.20 _	_1.52 _	_0.39 _		

## Discussion

Our study had shown the average duration of surgery for DHS with de-rotation screw was 48.8 minutes, which is longer than the average time required for PFN of 38.6 minutes. Li H et al. and others [13-16] also observed similar findings in their study. The significant difference between operative times is as a result of 1) smaller incision and less soft tissue dissection with PFN 2) easier and faster closure with PFN. Mean blood loss in group one was 312 ml, and group two was 108 ml. The difference between the two groups was statistically significant (p<0.000). Shen et al. have shown similar results in their studies. 

The mean Harris Hip Score in DHS with de-rotation screw group was 78.93 and in the PFN group was 82.67. These results were comparable to results obtained by Li H et al. and others [13-16]. It was found to be not statistically significant, and the long-term results of both groups were similar. Our study had shown no significant difference in postoperative hip function after union at six months in both groups. (P-Value> 0.05). All fractures in both groups had united by six months. One case of screw cut out and “Z” effect was seen in both groups. It is evident that postoperative complications were comparable in both groups. 

Limitations of the study: long-term follow-up in terms of restoration of pre-injury ambulatory status, mortality, and secondary arthritis may not be possible, and ethnicity of result cannot be stated due to small sample size, i.e., 15 cases in each group. 

## Conclusions

Both DHS with de-rotation screw and PFN are equally effective for the treatment of intertrochanteric fractures in the elderly as they allow equally good functional outcomes after fracture union. Based on the observations made in our study, we can safely conclude that PFN gives better results than DHS with de-rotation screw in intertrochanteric fractures in terms of the amount of blood loss during surgery, duration of surgery, early toe-touch weight-bearing, and Harris hip scores. There is no difference between the two modalities in terms of duration of hospitalization, fracture union, mortality and morbidity, and postoperative complications
